# Applying Ecological Momentary Assessment (EMA) to Understand Overweight and Obesity: A Systematic Review

**DOI:** 10.3390/jpm15110526

**Published:** 2025-11-01

**Authors:** Giada Rapelli, Chiara A. M. Spatola, Giulia Landi, Eliana Tossani, Silvana Grandi, Gabriella Martino, Gianluca Castelnuovo, Giada Pietrabissa, Roberto Cattivelli

**Affiliations:** 1 Department of Psychology, Catholic University of Milan, 20123 Milan, Italy; gianluca.castelnuovo@unicatt.it (G.C.); giada.pietrabissa@unicatt.it (G.P.); 2 Department of Clinical and Experimental Medicine, University of Messina, 98125 Messina, Italy; chiara.spatola@unime.it (C.A.M.S.); gabriella.martino@unime.it (G.M.); 3 Department of Psychology Renzo Canestrari, Alma Mater Studiorum University of Bologna, 40127 Bologna, Italy; giulia.landi7@unibo.it (G.L.); eliana.tossani2@unibo.it (E.T.); silvana.grandi@unibo.it (S.G.); roberto.cattivelli@unibo.it (R.C.); 4 Clinical Psychology Research Laboratory, IRCCS Istituto Auxologico Italiano, 20145 Milan, Italy

**Keywords:** obesity, overweight, Ecological Momentary Assessment (EMA), systematic review, Binge Eating Disorder (BED), psychological processes

## Abstract

**Background:** Obesity is a complex health issue influenced by various factors, including behavioral patterns that can be assessed more deeply in real time using Ecological Momentary Assessment (EMA), which can capture the moment in which a person experiences a situation or an emotion that could trigger an eating behavior. **Methods**: This systematic review synthesizes findings from 89 studies employing EMA to investigate obesity and overweight-related behaviors. The studies were identified through comprehensive searches across multiple databases and included peer-reviewed articles. The primary aim was to analyze how EMA contributes to understanding the temporal dynamics of eating behaviors, physical activity, and psychological factors associated with overweight and obesity. **Results**: Key findings indicate that EMA provides a nuanced understanding of real-time contexts influencing behaviors contributing to overweight and obesity. Studies consistently report that EMA captures fluctuations in eating habits, exercise routines, stress levels, and emotional states, elucidating the interplay between these factors and weight status. Methodological variations across studies included differences in EMA implementation (e.g., smartphone apps, electronic diaries), assessment frequency, and duration. These variances highlight the flexibility and adaptability of EMA in capturing diverse behavioral aspects relevant to obesity and overweight research. Moreover, the review discusses methodological challenges such as participant compliance, data integration, and real-time data interpretation in longitudinal analyses. **Conclusions**: In conclusion, EMA emerges as a powerful tool for exploring the complex, dynamic nature of overweight and obesity-related behaviors. Future research should focus on refining EMA methodologies, enhancing data analysis techniques, and integrating findings into personalized interventions aimed at reducing obesity effectively.

## 1. Introduction

The World Health Organization (WHO) defines obesity as a complex chronic disease characterized by excess fat deposits that can compromise an individual’s health and cause serious pathophysiological [1], psychological [2], and social [3] consequences for people of all ages and socioeconomic groups [4]. In 1997, the WHO formally recognized obesity as a global epidemic [4]. Over the past 40 years, its prevalence has grown dramatically, causing significant health and economic consequences [4]. According to the Non-Communicable Diseases Risk Factor Collaboration (NCD-risC), a network of health researchers that provides up-to-date data on non-communicable diseases in 200 countries around the world, the prevalence of obesity in individuals aged 20 years and over has increased from 1990 to 2022 in 188 out of 200 countries (94%) in women and in 199 out of 200 countries (99%) in men [5]. In 2022, the prevalence of obesity in Italy was around 17.3% in individuals aged 18 years and over [6].

According to Castelnuovo et al. [7], psychological factors play crucial roles in the successful long-term treatment of obesity. These variables include self-esteem, quality of life, stressful life events, eating disorders, mood disorders, anxiety, and personality traits, all of which show significant correlations with obesity [7,8,9,10,11]. The most studied psychological factor in the literature is certainly emotional regulation among the emotional eating theory, by which eating constitutes a coping strategy in response to states of emotional distress [12,13]. In fact, failed emotional regulation could cause impulsive eating behaviors such as overeating and binge eating [14], which can promote weight gain [15] and the manifestation of an obesity condition [11,16]. Possible causes of this phenomenon are poor interoceptive awareness [17], high levels of alexithymia [18], and a reduced stress response by the hypothalamic–pituitary–adrenal axis (HPA) [19]. For the management of obesity, integrating various interventions (i.e., dietetic, nutritional, physical, behavioral, psychological, and, if necessary, pharmacological and surgical ones) in a multidisciplinary context is recommended. Among the effective psychological treatments, Cognitive Behavioral Therapy (CBT) [7] and Brief Strategic Therapy [20] are traditionally recognized as the best-established treatments for Binge Eating Disorder (BED) and obesity. Despite the demonstrated effectiveness of digital interventions in obesity management [21], few approaches integrate real-time, context-aware assessment methods such as the Ecological Momentary Assessment (EMA). EMAs, Experience Sampling Methods, Ambulatory Assessments, and daily methods represent a methodological advancement that has significantly enhanced the study of psychological processes in recent years, enriching our understanding of previous knowledge in both the research methodology and in clinical psychology and psychotherapy [22] (Ecological Momentary Assessment (EMA) differs from related approaches in that it emphasizes repeated, real-time self-reports in naturalistic settings, whereas ambulatory monitoring incorporates physiological data and digital diaries often rely on less frequent, retrospective entries.) It enables researchers and clinicians to gather ecologically valid, in-depth data from individuals, contrasting with traditional methods that rely on retrospective self-reports. EMAs involve real-time, repeated measurements of an individual’s experiences, behaviors, and physiological responses over time, making it ideal for exploring within-person fluctuations and trajectories [23].

In recent years, interest in EMA within clinical psychology and psychotherapy has surged, bolstered by advancements in digital technologies, notably mobile phones. Real-time data capture allows for a nuanced understanding of contextual influences in natural settings, inaccessible through laboratory-based assessments. This contextual sensitivity is crucial for disorders like obesity, which is profoundly shaped by situational factors [24]. Furthermore, in clinical practice, EMAs allow clinicians and therapists to monitor patients’ behavior change (in terms of patients’ behaviors, thoughts, emotions, and environments) to assess the emerging themes among sessions and treatment processes, improving self-awareness, motivation, and patient engagement, and finally, to predict therapy outcomes and treatment efficacy [25].

Overall, EMAs aim to personalize models of psychopathology by acknowledging each individual’s unique traits and their interaction with varying contexts, emphasizing the dynamic nature of functional and dysfunctional states. The rise in EMA is driving the development of personalized models using intensive longitudinal data, revealing factors influencing adaptive or maladaptive behaviors [26]. Compared to retrospective self-report measures, EMA may be more effective in capturing the co-occurrence of symptoms and psychological processes over time, providing precise insights into the proximal antecedents and consequences of eating behavior. This may contribute to a better understanding of eating behavior, particularly obesity, in natural settings, potentially improving psychological treatments by identifying mechanisms of change [27]. Despite its advantages and growing use, a systematic review of EMA studies in overweight and obesity is still lacking, which this study aims to address. Thus, the purpose of this systematic review was to (1) examine the published EMA studies on participants who are either overweight or obese, (2) produce a summary of the scientific evidence among psychological predictors and outcomes investigated in EMA studies, (3) identify the strengths and weaknesses of these studies; and (4) provide empirically supported suggestions for future research and clinical practice.

## 2. Materials and Methods

The protocol of this systematic review was registered with PROSPERO (ID: CRD42024559628). Data extraction, critical appraisal, and qualitative synthesis were in line with established systematic reviews and qualitative synthesis methods [28]. This report follows the Preferred Reporting Items for Systematic Reviews and Meta-Analyses (PRISMA) statement [29] (Table S1).

### 2.1. Search Strategy

Searches were conducted in the following databases: PubMed, Scopus, PsycINFO, and Web of Science databases, between 1 and 10 July 2025. The two search strategies (one for obesity and one for EMA) combined key terms and Medical Search Headings (MESH) terms based on the patient problem (or population), intervention, comparison (or control), and outcome in accordance with the PICO [30] elements, as follows:

((ema) OR (“ecological momentary assessment”) OR (“mobile health”) OR (“experience sampling method”) OR (“ambulatory assessment”) OR (“personal digital assistant”) OR (“ambulatory monitoring”) OR (“real-time data capture”) OR (“real-time monitoring”) OR (“real-time interventions”) OR (“electronic diary”) OR (“repeated observations”) OR (“diary data”) OR (“time series”)) AND ((obes*) OR (“binge eating disorder”) OR (“overweight”)).

### 2.2. Inclusion and Exclusion Criteria

Articles were included if they (1) were published in English, (2) were original research articles, (3) employed EMAs, (4) reported at least one psychological primary outcome, (5) included adults with obesity, and (6) reported a mean of Body Mass Index (BMI) ≥ 25, as suggested by Center for Disease Control criteria. Studies were excluded if they (1) reported only biomedical data, (2) employed a quantitative approach different from EMA, (3) included children or adolescents, and (4) were not original studies (i.e., epidemiological studies, opinion or prospective studies, theoretical case studies, protocol studies, and a collection of previous samples). No limitations were imposed on gender, sample size, or ethnicity. Unpublished works and gray literature were not considered. The reference lists of all selected articles and retrieved systematic reviews were manually screened to identify any additional contributions for possible inclusion; however, none were found.

### 2.3. Study Selection

Following the search and exclusion of duplicates, two reviewers (authors GR and CS) independently assessed the eligibility of the articles, first by reviewing the title and abstract and then the full text, according to the inclusion criteria. Screening of titles and abstracts was performed using Rayyan [31], which facilitated blinded independent assessment and automatic detection of conflicts between reviewers. Disagreements were resolved by another reviewer (GP). Following Smith et al. [32], the review team included at least two persons with methodological expertise in conducting systematic reviews (GR and GP) and at least two experts on the topic under review (GP and RC). Searches of electronic databases identified 7069 reports. Of these, 3512 were duplicates, and 3453 records were excluded based on information from the title and abstract.

**Figure 1:**
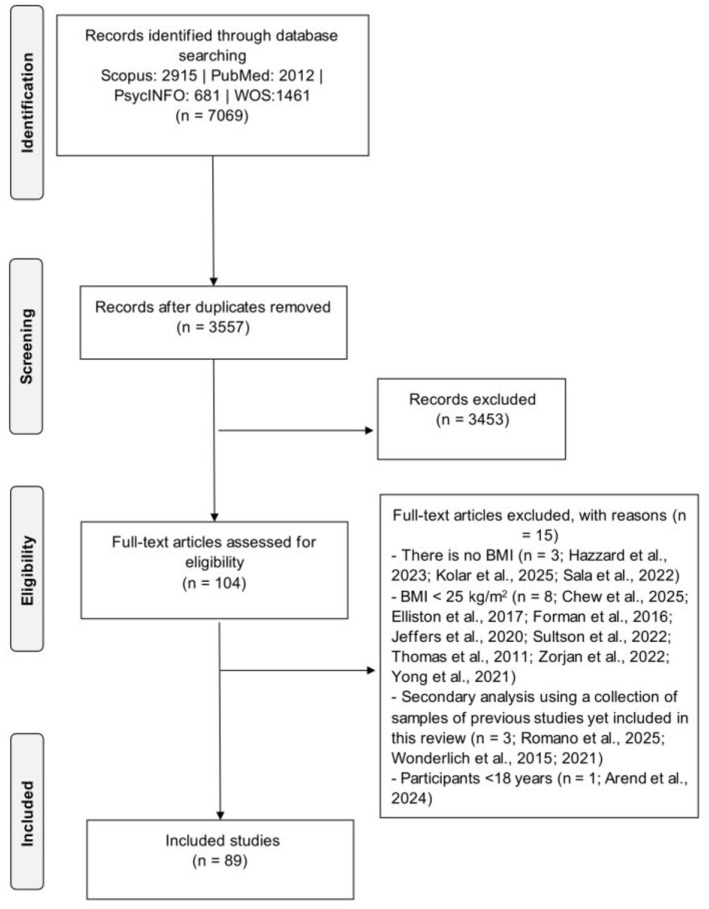
The flowchart of the study [33,34,35,36,37,38,39,40,41,42,43,44,45,46,47].

The remaining 104 records were evaluated for inclusion by reviewing their full texts, resulting in the inclusion of 89 articles. The flowchart presented in Figure 1 provides step-by-step details of the study selection.

### 2.4. Data Extraction and Synthesis

Two authors (GR and CS) independently extracted the following data from the included studies: (1) first author and year of publication, (2) country, (3) study aim, (4) EMA sampling frequency (daily, hourly, and multiple times per day), (5) time-based observations: frequencies per day/total number of days (sampling points), (6) number of participants (n), (7) age, (8) sex, (9) Race/Ethnicity, (10) Comorbidity, (11) BMI, (12) software or prompt delivery, (13) hardware, (14) data analysis, (15) predictors and measures, (16) clinical outcomes and measures, and (17) main findings.

They discussed any discrepancies and, if necessary, consulted a third author (RC) to reach a final decision. Extracted data were collated to produce a narrative summary of EMA studies in the context of obesity.

### 2.5. Quality Appraisal

As a specific quality appraisal tool for EMA studies is currently unavailable, a specific tool for this review, drawing on the previous literature [48,49], was employed. The quality appraisal tool included the following four criteria: (1) rationale for the EMA design; (2) whether an a priori power analysis had been conducted; (3) adherence to the EMAs; and (4) treatment of missingness. In line with the Effective Public Health Practice Project quality assessment tool [50], the four criteria are rated as ‘Strong’, ‘Moderate’, or ‘Weak’. Finally, an overall study quality rating for each study was produced. The quality appraisal was performed by one reviewer from the author team, with 20% double-checked by a second reviewer. Discrepancies were resolved through discussion with another reviewer and consulting the other team members if needed. The inter-rater reliability was not calculated.

### 2.6. Data Synthesis

A narrative (descriptive) synthesis was conducted to summarize the theoretical and methodological aspects of the EMA studies. To notify overlapping samples across included studies at the time of data extraction, this approach was followed: (i) two reviewers (GR and CS) flagged studies with identical sample sizes and mean ages; and (ii) checked the author list for overlaps in co-authorship. Where (i) and (ii) were satisfied, studies were coded as having an overlapping sample. Where an overlap in co-authorship was not identified, the full texts were further checked.

Next, where sample sizes and mean ages were very close but not identical, the articles were further screened to check for overlapping samples. Similar papers were identified with the same color in Supplementary Material File S1.

## 3. Results

### 3.1. Study Characteristics

Details of the 89 included papers are provided in Supplementary Material File S1.

The selected articles were published from 2001 [51] to 2025 [34,39,40,52,53,54,55,56,57,58,59,60,61] and were conducted in the USA (n = 67), Netherlands (n = 5; [54,55,62,63,64]), Switzerland (n = 4; [65,66,67,68]), Germany (n = 4; [60,69,70,71], Australia (n = 2; [72,73], Canada (n = 2; [74,75]), United Kingdom (n = 2; [57,76]), Romania (n = 1; [77]), Poland (n = 1; [78]), and Belgium (n = 1; [79]). Four studies employed a randomized trial [80,81,82,83], two studies employed a Randomized Control Trial (RCT) design [84,85], and twelve studies employed a comparative study on several populations: lower body fat vs. higher body fat (n = 1; [76]), healthy-weight vs. overweight/obesity population (n = 5; [62,63,78,80]), obese BED vs. obese non-BED vs. non-obese controls (n = 1; [86]), obese BED vs. obese non-BED (n = 2; [87,88]), BED population vs. population with bulimia vs. healthy controls (n = 1; [69]), obese black vs. obese white (n = 1; [89]), participants who smoke vs. overweight and BED participants (n = 1; [90]), and participants with anorexia vs. bulimia vs. BED (n = 1; [91]).

### 3.2. Description of Participants

The selected contributions included a total of 6917 participants with overweight or obesity of both genders (4049 males and 2831 females), while four studies did not report the participants’ gender [63,78,86,88]. The sample size varied from a minimum of 12 [92,93] to a maximum of 363 participants [94].

The mean age of all participants is 43.18 years, and the range of years varied from 18 to 85. One study did not report the age of participants [95]. The majority of studies include Caucasians or Whites, and 17 studies did not report race and ethnic characteristics. Among the participants, 2152 (31%) met the criteria for BED. Because individuals with BED comprise a significant subset of individuals with overweight and obesity [11,96], these individuals were included in this review to enhance the representativeness of the sample and the generalizability of the findings. Other included comorbidities were Attention-Deficit/Hyperactivity Disorder (ADHD) [60] and depression [97], and among physical diseases, type 2 diabetes mellitus, Hypertension, Osteoarthrosis, Gastroesophageal Reflux Disease, Dyslipidemia [55], and general pain conditions (i.e., chronic pain, arthritis, migraine, or neuropathy) [98] were included. One study was conducted with patients undergoing metabolic and bariatric surgery [52].

### 3.3. Description of Study Aims

The included studies collectively investigated the psychological, emotional, and contextual drivers of eating behaviors, especially binge eating (BE), dietary lapses, and weight regulation, across various populations. A major theme involved identifying momentary and habitual triggers, such as Negative Affects (NAs), stress, and interpersonal tension that predict disordered eating patterns.

For example, Ambwani et al. [99] and Dougherty et al. [56] explored how negative emotions and interpersonal stress contribute to BE, while Schaefer et al. [100] and Goldschmidt et al. [87] examined how emotional reinforcement (e.g., reduction in NA) maintains binge behavior. Studies like Keating et al. [74] and Parker et al. [101] emphasized the roles of emotional dysregulation and daily emotional variability in predicting Loss-Of-Control (LOC) eating.

Several studies explored habitual processes and how they relate to behavior persistence, such as Dougherty et al. [102], which links habitual control over BE to its maintenance. Chwyl et al. [103] and Clark et al. [104] looked at how everyday activities and social contexts influence dietary lapses and eating behavior.

In terms of treatment and intervention, Bartholomay et al. [84] and Peterson et al. [85] compared cognitive and emotion-focused therapies to reduce BE. Coffman et al. [105] and Godfrey et al. [81] examined how acceptance-based treatments affected stress-related eating and physical activity. Mason et al. [106] and Sagui-Henson et al. [107] applied mindfulness-based mobile interventions to target cravings and emotional eating.

The impact of weight stigma and internalized bias was examined by Carels et al. [95] and Olson et al. [98], showing links to emotional responses, pain, and disordered eating. Forester et al. [108] and Nechita et al. [77] highlighted the role of shame and self-conscious emotions in perpetuating maladaptive eating behaviors.

Special populations, such as post-bariatric surgery patients [52,55], older adults [79], and those with food insecurity [57], were studied to understand how contextual vulnerabilities modify the risk and maintenance of disordered eating.

Finally, studies like Srivastava et al. [93,109] and Wonderlich et al. [110] delved into how body dissatisfaction, feeling fat, and trait-level impulsivity contribute to BE symptoms across time.

### 3.4. Description of EMAs

Regarding the methodological characteristics of EMA applied among the included studies, three types of sampling have been identified. On the one hand, six studies implemented a daily diary, which typically entails one end-of-day report [51,81,83,95]. On the other hand, other studies comprise different numbers of daily assessments and diverse types of prompt contingencies. The duration of the EMA ranged between 2 [69] and 182 days [83]. The most frequently implemented study duration was 14 days, applied in 32 studies. The frequency of assessments per day ranged from 2 [73,80,111] to 32 [69]. The most implemented frequency of daily assessment was six, implemented in 27 studies. The total number of sampling points ranged from 10 [111] to 420 [112]. The most commonly used EMA sampling strategy involved fixed daily prompts, applied in 78 studies (87.64%), most often before, during, and/or after each eating episode. Among these, more than half of the studies (41/78, 52%) used a multiple sampling method incorporating an event-contingent design with scheduled random prompts. Furthermore, only five studies applied an event-contingent design [72,76,86,113,114]. In these cases, the participant was asked to voluntarily and autonomously complete the assessment (after appropriate training) at various times or situations: each time food wanting is perceived regardless of whether the participant is going to eat or not [76], every time the participant experiences weight stigma [114,115], every time the participant eats something [86], after each binge eating episode [116] or snacking episode [79] or purging [102], and every time the participant reports dietary temptation and lapse [72]. Finally, two studies did not report the frequency of assessments per day clearly [93,103].

Regarding the delivery modalities, the most used hardware was the participants’ smartphone, while the most used software platforms were ReTAINE [52,75,80,84,85,94,117,118,119], LifeData [58,59,98,112,120,121,122,123], Pendragon [67,68,99], Qualtrics [73,107], Paco [103,124], SurveySignal [56,102], the SEMA3 app [53], or an app/website specifically designed for study aims in most cases. Two studies declared the use of a paper-and-pencil approach to the daily diary [51] and to the weekly assessment [113], and twenty-three studies did not mention how they delivered the EMA assessments.

Regarding the data analysis, approximately 30% of the studies used Multilevel Modeling to account for the nested nature of the recruited data. Twenty-six studies (29.21%) applied a General Linear Model (GLM) or Generalized Linear Mixed Model (GLMM). Eighteen studies used Generalized Estimated Equations (GEE; 18/89; 20.22%), ten studies calculated ANOVA models or t-tests, three studies ran a logistic regression, two studies applied a cross-lagged model, two studies applied a correlation analysis, and one study applied a decision tree analysis.

### 3.5. Description of Measures

#### 3.5.1. Clinical Measures

Thirteen studies included at least one clinical measure. In particular, the most frequently used outcomes in six studies were weight loss and the percentage of weight loss maintenance [65,73,81,92,106,112]. This clinical variable was followed by the calculation of BMI in three studies [68,114,125]. Furthermore, two studies assessed caloric intake [87,88]. The last study used the Nutritional Data System for Research (NDS-R) [80]. One study measured the bite count, duration, and rate (seconds per bite) [112]. One study used Actigraph to assess Moderate-to-Vigorous Physical Activity (MVPA), light activity, and sedentary time [126].

#### 3.5.2. Predictor Variables

The most frequent predictor was the mood or Positive (PA) and Negative Affect (NA), included in 35 studies using the Positive and Negative Schedule (PANAS) [52,56,87,97,99,117,125,127,128,129,130], the Feeling Scale [131], the Mood Assessment Inventory (MAI) [68], and the ad hoc scale or Visual Analog Scale (VAS) items [51,55,69,71,90,107,111,114,123,129,132,133]. Other predictors connected to the emotional domain were also assessed. The second most frequent predictor was emotional regulation using the Difficulties in Emotion Regulation Scale (DERS) [74,101,117], the Emotion Regulation Skills Questionnaire (ERSQ) [70], the Emotion Regulation Questionnaire (ERQ) [70], the Perseverative Thinking Questionnaire (PTQ) [70] and the Daily Habits Questionnaire (DHQ) [134]. One study [110] measured a specific aspect of emotional regulation—state and trait urgency, defined as the tendency to act rashly when experiencing negative emotions. Another study measured the emotional state by asking participants how often they experienced emotions [64]. Guilt was measured in five studies using the Positive and Negative Affect Schedule—Expanded Form (PANAS-X) [100,119], the State Shame and Guilt Scale (SSGS) [73], and ad hoc items/scales [111,133]. Shame was assessed in four studies using the SSGS [73], the Emotional Eating Questionnaire (EES) [77], and using ad hoc items/scales [111,135]. Depression was measured in two studies with the Beck Depression Inventory (BDI) [125] and the Depression Anxiety Stress Scales (DASS-21) [74]. Anger was assessed in one study using ad hoc items [136]. Physical anhedonia was measured in one study [80] using the Physical Anhedonia Scale (PAS). Trait and state impulsivity were assessed in one study [60] using the Momentary Impulsivity Scale (MIS) and the Urgency Premeditation Perseverance and Sensation Seeking Impulsive Behavior Scale (UPPS-P), respectively.

Stress was measured as a predictor in 11 studies with the Perceived Stress Scale (PSS) [60,80], the PANAS [87,105], and ad hoc items/scales [54,69,72,90,125]. One study [56] measured the impact of interpersonal stress on the maintenance of BE and purging using items from the Daily Stress Inventory (DSI), while another study [58] measured the frequency and impact of weight-related distress on eating and BE using ad hoc items.

Sleep outcomes in terms of sleep quality, duration, and sleepiness/fatigue were measured in three studies using the Total Sleep Time (TST) [137] and ad hoc items [92,138].

General self-abilities and psychological processes not strictly related to eating were evaluated in 10 studies. In particular, Thøgersen-Ntoumani et al. [73] measured state and trait self-compassion using the Self-Compassion Scale–Short Form (SCS-S). Hagerman et al. [139] also assessed self-compassion in terms of self-kindness, common humanity, and mindfulness using ad hoc items. Mckee et al. [72] measured future self-efficacy and the ability to think in the long-term and about the importance of goals using ad hoc items. Schumacher et al. [140] measured self-attitudes including self-forgiveness, self-regard, and self-efficacy using an ad hoc scale. Sala et al. [124] measured mindful awareness, willingness, and values clarity using ad hoc single items adapted from the PMS and the Food and Acceptance Action Questionnaire (FAAQ). Schumacher et al. [140] measured self-criticism with ad hoc items. Mason et al. [128] measured self-discrepancy using ad hoc items. Svaldi et al. [70] measured suppression and rumination using ad hoc questions. A study [75] measured the cognitive and behavioral skills learned after a multidisciplinary 6-to-8 weeks CBT-based program by asking participants to indicate what strategies they used from the following items: distracting activities, mechanical eating, delay, planning ahead, social support, riding the wave/sitting with emotions, activities to produce an alternate emotion, changing the environment, coping statements, opposite action, observing and labeling emotions, self-soothing, and made the environment safer (i.e., stimulus control). One study [54] measured the role of coping self-efficacy behaviors and the recovery self-efficacy in predicting dietary habits using ad hoc items based on the health-specific self-efficacy scales.

Regarding self-abilities and personal skills related to eating, Latner et al. [65] measured self-efficacy to control eating behavior with the Weight Efficacy Lifestyle Questionnaire (WEL) and coping with high-risk eating situations using the Hypothetical High-Risk Task (HHRST). Similarly, Carels et al. [113] measured the coping response during temptations or lapses by asking participants to indicate it from the following items: “Removed myself from the situation”, “Distracted myself”, “Talked to a group member for advice or comfort”, “Talked to a family member for advice or comfort”, “Talked to a friend for advice or comfort”, “Encouraged myself”, “Meditated/relaxed”, “Engaged in spiritual activities”, “Exercised”, “Thought about the benefits associated with dieting”, “Thought about the benefits associated with being healthy”, “Thought about the negatives associated with not dieting”, “Thought about the negatives associated with being unhealthy”, and “Other”. Hagerman et al. [111] measured the confidence in and motivation for weight control using ad hoc scales. Furthermore, Hagerman et al. [111] measured the perceived control over weight management behavior with ad hoc items. Crochiere et al. [141] measured the confidence in meeting dietary goals and planned food intake. One study measured the pre-meal and/or post-meal locus of control using ad hoc items [87]. The ability to detect the level of hunger was assessed in seven studies using ad hoc items [72,87,90,92,125,129,132]. One study [53] measured the weight-related vigilant coping, such as the ability to detect and avoid stigmatization via hypervigilance and behavior monitoring applied by people who think they may be stigmatized using the Williams Heightened Vigilance Scale (WHVS).

In contrast, there were 20 studies that measured problematic eating behaviors or negative thoughts/behavior regarding the body. In particular, Kornacka et al. [78] measured repetitive Negative Thinking with the PTQ and emotional eating with the Three-Factor Eating Questionnaire (TFEQ). Ad hoc items were used by Srivastava et al. [93] to measure body dissatisfaction, and in the following study in 2024, the same authors [142] used ad hoc measures to assess feelings of fatness. Hilbert et al. [69] measured negative cognitions on food/eating and body image using ad hoc items. Mason et al. [126] measured body satisfaction and eating-related rumination using the Ruminative Response Scale for Eating Disorders Brooding subscale (RRSED-B). Rancourt et al. [143] measured weight-focused social comparisons and their targets using ad hoc items. Also, MacIntyre et al. [118] measured body social comparisons and external pressure for thinness using ad hoc items. Also, eight other studies measured food cravings or addiction using ad hoc items [97,117,119,125,129,132]. The study by Kalan et al. and Li et al. [120,121] measured food addiction using items from the Yale Food Addiction Scale (YFAS) and binge eating with the Eating Pathology Symptoms Inventory (EPSI). Weight stigma was assessed in four studies; in particular, Olson et al. [98] and Vartanian et al. [114] measured experienced weight stigma using the Stigmatizing Situations Inventory (SSI) and another study using ad hoc items [53]; furthermore, Olson et al. [98] and Carels et al. [95] measured the internalized weight bias using the Weight Bias Internalization Scale (WBIS). One study measured the urge to deviate from an eating plan using ad hoc items [141]. One study measured binge eating episodes and the likelihood of binge eating in the next 4 h using the TFEQ [90]. Similarly, Forester et al. [108] measured binge anticipation in terms of the planning and inevitability of the event using ad hoc items. Snacking episodes (such as the type and portion of the snack) were analyzed in the study by Cnuddle et al. [79] using ad hoc items. Dougherty et al. [102] measured the LOC in BE episodes using ad hoc 5-point Likert items. The dietary temptation was measured in one study using ad hoc items [72].

Some studies measured the contextual factors. One study [55] measured the contextual factors and activity more generally in predicting eating behaviors in people after Metabolic Bariatric Surgery (MBS). The presence of others who would be suitable for social modeling was measured in 11 studies using ad hoc items or multiple-choice questions [51,64,70,72,79,90,97,104,125,130,133,144]. The location where the participant is during the eating behavior was measured in 10 studies using ad hoc items or multiple choice questions [51,64,70,90,97,104,122,130,133,144]. The presence of delicious/tempting food in the location where the participant was was measured in four studies [90,92,129,132]. Two studies assessed interpersonal problems in terms of arguing with someone, feeling rejected, feeling lonely, wishing their relationships were better, and wishing to have more friends using ad hoc items [118,129]. One study measured loneliness using ad hoc items [59]. The consumption of alcohol was measured in four studies with ad hoc items [90,92,125,132]. One study assessed the participants’ attachment using the Close Relationships Scale (ECR) [74]. The activity that the participant was performing was measured in five studies [51,132,133], as well as watching TV [129,132] and the access to food advertising [113].

One study [84] assessed dietary restrictions with ad hoc items, separately examining meal skipping and the longest time without eating. Connected to this point, one study [145] measured the food intake by asking participants to note whether they had consumed the following: (a) sweets (e.g., chocolate, cookies, and cake), (b) fast food or fried food (e.g., fries, chips, and pizza), (c) sugar-sweetened beverages (e.g., soda, sweetened tea/coffee), and (d) fruits or vegetables.

Roordink et al. [64] measured the perceived presence of social support, perceived descriptive and injunctive norms, and perceived social pressure with ad hoc single items.

One study [80] measured the community-level assessment of subjective social status using the MacArthur Scale of Subjective Social Status. Two studies [57,80] measured the economic insecurity of buying food, such as the experiences of hunger, meal-skipping, and consuming an imbalanced diet in the last 12 months as a result of the unaffordability of food using the USDA Household Food Security Survey Six-Item Short-Form module (FSQ).

Two papers [67,68] also included the feasibility of and reactivity to EMA using EXQ.

One study measured the perceived physical state using an ad hoc VAS [55].

One study measured the perceived hunger using ad hoc items [54].

#### 3.5.3. Outcomes Variables

Thirty-eight studies assessed the frequency and characteristics of eating behavior and binge eating episodes in terms of overeating and the loss of control, according to the Diagnostic and Statistical Manual of Mental Disorders (DSM) diagnosis, using the Overeating Monitoring Questionnaire (OMQ) [99] for overeating or the Eating Disorder Examination Questionnaire (EDE-Q) [53] or using ad hoc items asking the participant to report when the overeating occurs during the day, together with the feeling of LOC after specific training [52,55,56,58,59,69,70,74,75,82,84,85,87,88,89,90,97,100,101,102,104,108,109,116,117,119,120,121,122,123,125,126,127,128,135,144]. Some authors assessed other problematic eating behaviors. Four studies [56,75,77,102] measured purging with ad hoc items. Two studies measured snacking behavior with ad hoc items [78,79]. Srivastava et al. [109] and Nechita et al. [77] assessed the presence of several eating disorder behaviors (i.e., BED, self-induced vomiting, laxative misuse, diet pill misuse, compensatory exercise, dietary restraint, and actual restriction) multiple times per day.

Nineteen studies assessed the frequency and/or severity of temptations and lapses in dietary or exercise behavior with ad hoc questions [51,54,58,65,72,92,103,105,112,113,123,124,129,132,139,140]. Furthermore, Latner et al. [65] measured coping with lapse using ad hoc questions, and conversely, Thøgersen-Ntoumani et al. [73] measured the negative reaction to lapse occurrence using items adapted from the Dichotomous Thinking in Eating Disorders Scale (DTEDS).

A group of included studies investigated some eating attitudes and behaviors. Four studies assessed the level of appetite using the Momentary Appetite Scale (MAS) [120,121] or ad hoc questions [57,118], and two other studies measured eating behavior in the absence of hunger [130] or the desire to eat with an ad hoc single VAS item [57]. Mason et al. [146] used the Palatable Eating Motives Scale (PEMS) to explore the motives for eating tasty food (social, conformity, enhancement, and coping motives). Also, another study [76] explored the attitude toward healthy food using both the Explicit Attitudes Towards Healthy Food Questionnaire and the Implicit Association Task (IAT), showing images of unhealthy vs. healthy foods. Furthermore, Mason et al. [106] also assessed reward-driven eating using the Reward-based Eating Drive Scale (RED). One study [57] assessed the desire to eat with an ad hoc single VAS item, and one study assessed eating as a strategy for coping using the Motivation to Eat Scale [53].

Eight studies assessed food cravings/food addiction using the Food Craving Questionnaire (FCQ) [76,106], or an adapted version of the Yale Food Addiction Scale 2.0. [59], or using ad hoc items [63,97,106,107,123].

Five studies [77,95,114,115,147] assessed weight stigma and associated correlates (e.g., target, expression of stigma, response, eating or exercise activity reactions, suppression of response, location, and presence of others) using ad hoc questions. Connected to this theme, one study [134] measured the fear of weight gain and the feeling of being fat using the Daily Habits Questionnaire (DHQ), and Carels et al. [114] measured body appreciation using the Body Appreciation Scale-2 (BAS-2). Conversely, Kalan et al. [120] and Li et al. [121] measured body dissatisfaction using ad hoc items.

Seven studies assessed the mood state, with five using the PANAS [75,94,108,120,121]. Furthermore, Kalan et al. [120] and Li et al. [121] also measured impulsivity with a subscale of DERS. One study assessed the emotional state with the PHQ and anxiety with the GAD [89], and the last one [62] measured emotional eating with the DEBQ and the EES.

Four studies assessed physical activity and exercise behavior. Williams et al. [83] measured the duration of exercise behavior. Godfrey et al. [81] measured physical activity intention and physical activity behavior with ad hoc items. Imes et al. [137] measured the total step count and sedentary behavior with the MVPA and Actigraphy. Also, Seiferth et al. [71] measured physical activity using device-based measures. Carels et al. [115] measured physical activity intention and behavior using ad hoc items.

One study measured food intake and food availability using ad hoc items [60].

One study [116] measured drinking behavior with ad hoc questions.

Finally, one study [98] explored bodily pain with the MOS-Short Form-36 (SF-36) as well as the pain condition with ad hoc items.

### 3.6. Description of Main Findings

**Figure 2:**
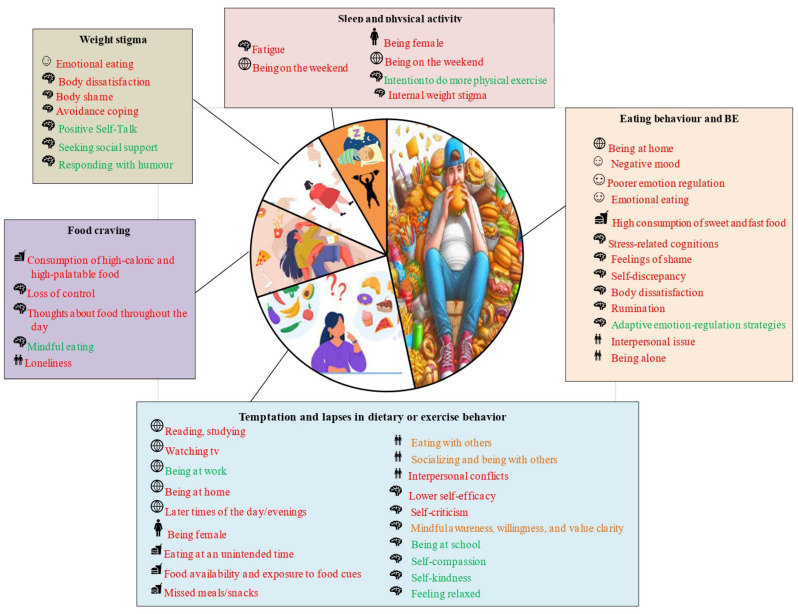
Summary of the findings. Legend. 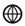 = contextual factors; 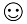 = emotional factors; 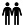 = relational factors; 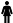 = gender-related factors; 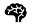 = psychological or cognitive processes; and 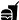 = food-related factors.

The principal findings of the included studies are delineated into distinct domains, reflecting their specific contributions to the field and primary areas of inquiry. Figure 2 presents a graphical synthesis of these findings: the relative size of each area denotes the frequency with which themes were identified across studies. Within the boxes, risk factors are indicated in red, protective factors in green, and factors with inconclusive associations in yellow.

#### 3.6.1. Eating Behavior and BE Episodes

Several studies [77,99,100,125] demonstrated that momentary NAs influence episodes of BE and LOC. For example, Kerver et al. [52] showed that a higher NA predicted more severe LOC eating after MBS; while Kuipers et al. [55] showed inconclusive results on the relationship between NAs and PAs and problematic eating behavior. Importantly, average levels of NAs across the sampling period were not related to BE [99]. In contrast, Mason et al. [128] found that when PAs were above average, men reported greater BE severity within the next two hours, whereas an elevated NA was associated with reduced BE severity. The consumption of sweets and fast food also increased BE severity [122], a finding confirmed by Smith et al. [123], who showed that greater instability in the PA predicted higher overeating and LOC. In a post-meal context, Goldschmidt et al. [87] reported that, among individuals with BED, a higher LOC was associated with greater post-meal NA, regardless of food intake. Conversely, in obese individuals without BED, a greater kilocalorie consumption predicted lower post-meal NAs. Dougherty et al. [102], further found that a longer BE duration and greater habitual control over BE were linked to smaller reductions in NAs following episodes. The study by Wilkinson et al. [116] showed that anxiety was highest on days with both BE and heavy drinking, while guilt increased prior to BE and sadness decreased afterwards. Srivastava et al. [109] identified “feeling fat” as a proximal predictor mediating the relationship between guilt and BE. Cravings were another consistent predictor: LOC episodes were often preceded by cravings [97,130], and craving-induced BE was partially mediated by guilt [119]. Parker et al. [101] observed that poor emotion regulation (ER) predicted LOC, while adaptive regulation strategies were protective [70]. Additionally, LOC was 3.6 times more frequent in BED than non-BED individuals, even after accounting for the affective state and caloric intake [88].

Emotional eating strongly correlated with BE, mediating the link between depression and BMI [81,124]. Depression and low mood were consistently associated with higher NAs, emotional eating, and BE [69,74,125]. Bulimia patients had more frequent negative food-related and stress-related cognitions during BE than individuals with BED [69]. BE episodes were associated with lower pre-episode hunger [125,144] and higher post-episode shame [125] and depression [69]. Also, Schaefer et al. [100,127] found that, following BE, levels of NAs and guilt significantly decreased, and the PA stabilized, suggesting that BE may temporarily alleviate negative emotions. These findings partially support the affect regulation model. Mason et al. [135] supported this theory through an intervention study that showed that patients who experienced BE as distressing, rather than reinforcing, benefited more from emotion-focused therapies such as ICAT-BED.

Additional work highlighted cognitive vulnerabilities: rumination and self-discrepancy [126] predicted BE, while body dissatisfaction influenced the probability of experiencing BE [93]. Shame also played a key role: greater shame predicted more disturbed eating behaviors across diagnostic groups [77]. Anticipatory planning of BE reliably forecasted future episodes and mediated increases in shame [108]. In contrast, positive thoughts and intentions toward healthy snacking were protective [79].

Regarding contextual factors, Forester et al. [117] identified that the risk for BE and overeating peaked around 5:30 p.m., with additional peaks at 12:30 p.m. and 11:00 p.m. In contrast, LOC eating without overeating was more likely to occur before 2:00 p.m. The risk for BE, LOC-only eating, and overeating only did not vary significantly across different days of the week. NAs did not show a consistent daily pattern but decreased slightly on weekends, while PAs decreased in the evenings and showed a smaller decrease on weekends. Patterns of food cravings and difficulties in emotion regulation mirrored the pattern of BE, with peaks around mealtimes and the late evening.

Additionally, Ambwani et al. [99] reported that interpersonal issues moderate the association between NAs and BE. Keating et al. [74] partially supported this finding, noting that the link between attachment anxiety and BE is moderated by emotional dysregulation. MacIntyre et al. [118] showed that interpersonal problems, body–social comparisons, and external pressures for thinness predicted appetite 4 h after. Also, Dougherty et al. [56] confirmed that individuals with habitual purging may be vulnerable to engaging in purging when they are experiencing high levels of interpersonal stress.

At home, being alone was significantly associated with less frequent eating and drinking, while at work, being alone was significantly associated with a greater frequency of eating or drinking. At home eating was most frequent in the afternoon and evening hours [104]. Also in Mason et al. [122], BE symptoms were lower when at work/school and other locations vs. at home and were higher at restaurants compared to at home.

#### 3.6.2. Temptation and Lapses in Dietary or Exercise Behavior

Lapses and temptations were strongly correlated, with lapses increasing alongside dietary temptation intensity [72]. Higher cravings predicted concurrent but not future lapses [124]. On average, participants with obesity and overweight reported 3.47 lapses per week [129]. Among lapse types, only eating at an unintended time was predictive of poorer weight loss outcomes [92]. The most common predictors of lapses were food availability and exposure to food cues/snacks [72,79,92], followed by missed meals/snacks and watching TV [92].

Temptations and lapses were more frequent at home than at school or work and were linked to greater hunger, reduced satiety, and feelings of sadness, stress, boredom, and loss of control [36,40]. Temptations and lapses in dieting and exercise behaviors often occurred while reading, studying, or during interpersonal conflicts, whereas lapses were more likely when eating with others. Both increased the risk of future lapses, creating a self-perpetuating cycle. Conversely, engaging in alternative activities such as chores, cooking, or prayer reduced lapse risk [103]. Relaxation and higher coping self-efficacy also protected against lapses [54], while vigilant coping predicted restrictive dieting [53]. Socializing and the presence of others often increased lapse risk [72,103], but the results were inconsistent [64].

Several other factors were associated with the increased odds of lapses. Being female [105] and later times of the day/evenings [64,72,105,112] were significantly related to higher odds of lapses. Stress was positively and significantly related to the odds of lapses. Coffman et al. [105] demonstrated that an ACT-based program, compared to standard behavioral treatment, moderated the effect of stress on dietary lapses. Lapses appeared to trigger a vicious cycle: lower self-efficacy, self-criticism, and negative self-regard increased lapse frequency, while lapses in turn worsened mood, guilt, shame, and confidence in weight control [65,113,140]. According to Hagerman et al. [111], participants who gained weight reported more negative moods, more guilt/shame, and a lower confidence in weight control.

Among protective psychological resources that could buffer negative reactions after a lapse, self-compassion and self-kindness improved the perceived self-control over weight management [111]. Furthermore, self-compassion was positively related to intentions and self-efficacy to continue dieting and fewer negative affective reactions to lapses [73]. Similarly, mindful awareness, willingness, and value clarity reduced cravings and lapses [124].

#### 3.6.3. Food Craving

Kalan et al. [120] declared that food addiction is different from BE, suggesting that addiction symptoms may reflect greater binge severity, emotional arousal, and impulsivity. Li et al. [121] added that food addiction severity moderated the link between food cue exposure and subsequent BE symptoms. Roefs et al. [63] reported that overweight participants experienced more frequent high-caloric and high-palatable (HCHP) food cravings during non-eating moments than normal-weight participants, who instead reported more staple food cravings during meals.

Alabduljader et al. [76] observed that individuals with obesity reported fewer “wanting” events than healthy peers but anticipated stronger positive reinforcement from eating. Healthy participants scored higher on food liking, whereas those with obesity experienced more loss of control and stronger emotional responses during cravings. Similarly, Boh et al. [62] confirmed that healthy participants showed a greater restraint and indulged less, while participants with obesity had more frequent cravings, food-related thoughts, and more negative emotions when resisting cravings. Other findings highlighted moderators. Adults with ADHD were not at particular risk for stress eating [60]. LOC episodes were followed by heightened post-episode cravings [125]. Interventions targeting cravings through mindful eating proved effective: Mason et al. [106] and Sagui-Henson et al. [107] reported significant reductions in trait cravings, craving-related eating, and overeating, with effects persisting at follow-up. These interventions also weakened the association between negative moods and cravings. By contrast, an exercise program showed no effect on cravings compared to the controls [82]. Finally, loneliness predicted higher levels of food addiction [59].

#### 3.6.4. Weight Stigma

Evidence on the prevalence of weight stigma is mixed. One study reported only eight stigma episodes in a week [147], whereas Vartanian et al. [114] found that 91% of participants reported at least one episode over a two-week period. Furthermore, Internal Weight Bias (IWB) [95,115] was strongly related to both coping responses, body appreciation, eating behaviors, and the avoidance of exercise. Positive and NAs mediated many of the associations. Positive self-talk correlated with lower emotional numbness, whereas avoidance coping was associated with greater depression, shame, and unhappiness [115]. Participants reporting only an internal stigma showed significantly higher negative self-talk than those reporting external or environmental experiences of stigma [115]. Importantly, stigma prevalence was unrelated to BMI or demographics such as sex, age, income, or ethnicity [114]. Stigma was most often expressed through verbal comments, through body language/gestures, or multiple modalities by strangers, partners, friends, and parents. Almost half of the stigma experiences occurred at home or in public places, and stigma from strangers produced more negative effects than stigma from close relatives or the media. IWB also predicted body dissatisfaction and maladaptive behavior. Body shame prospectively increased the risk for BE, body-checking, and excessive exercise, while shame around eating predicted BE and body-checking [77]. Momentary IWB was associated with physical discomfort (pain, aches, and muscle soreness) at the same and later time points [98]. Weight-related stress predicted greater body dissatisfaction [58].

#### 3.6.5. Sleep and Physical Exercise

Manasse et al. [138] reported that morning fatigue and sleep quality were moderately negatively correlated, while sleep quality was positively correlated with sleep duration. Fatigue was negatively correlated with sleep duration. Poor sleep quality predicted later maladaptive exercise behaviors, and a shorter sleep duration was linked to compensatory purging. Imes et al. [137] reported that participants engaged in more MVPA on weekdays than on weekends. Regarding sleep characteristics, sleep time was shorter on weekday nights, though participants had fewer awakenings and less Minutes of Wake After Sleep Onset (WASO) compared to weekends. No significant difference was found in sleep fragmentation or sleep efficiency between weekday and weekend nights. Greater TST and being on the weekend were associated with less sedentary behavior, but neither TST nor WASO predicted the next-day step count or MVPA. Each additional 60 min of TST corresponded to 19.2 fewer sedentary minutes the following day. Being a female, having a higher BMI, and weekends were associated with less MVPA. More daytime sedentary time was associated with less WASO, fewer awakenings, and lower TST.

Intervention studies highlighted psychological influences on activity. Williams et al. [83] demonstrated that self-paced exercise indirectly influenced subsequent exercise duration/latency through positive affective responses. Godfrey et al. [81] found that an Acceptance and Commitment Therapy (ACT)-based intervention improved the intention to do more physical exercise compared to standard care; both groups increased MVPA, but in the ACT group, weight loss was partially mediated by stronger exercise intentions. Carels et al. [95] observed that individuals with a higher internal weight stigma reported greater urges to avoid exercise. Finally, Seiferth et al. [71] supported a bidirectional association between physical activity in daily life and energetic arousal: being more physically active in the prior 15 min increased energetic arousal and decreased calmness, which in turn predicted a greater PA in the following 15 min.

### 3.7. Quality Appraisal Results

The quality ratings are summarized in Table 1. Overall, studies generally received a ‘Weak’ rating (35/89; 39.32%) for Quality 1 (i.e., the rationale for the EMA design provided), a ‘Weak’ rating (70/89; 78.65%) for Quality 2 (i.e., no a priori power analysis had been conducted), a ‘Strong’ rating (38/89; 42.69%) for Quality 3 (i.e., an average adherence rate of at least 80% to the EMA protocol), and a ‘Weak’ rating (63/89; 70.78%) for Quality 4 (i.e., no analysis of EMA missingness or controlling for potential missing mechanisms).

## 4. Discussion

The reviewed studies highlight the complex dynamics of eating behavior, including appetite regulation, eating in the absence of hunger, and BE episodes. These findings align with the broader literature that suggests the role of psychological factors such as NAs, PAs, and emotion regulation, as well as cognitive and behavioral strategies and skills in eating behaviors associated with overweight and obesity.

For example, the findings of Goldschmidt et al. [87,125,130] emphasize that negative emotions and LOC often trigger BE episodes. This is consistent with the affect regulation model of BE, which suggests that individuals engage in BE as a way to manage or alleviate negative emotional states. However, some studies [100] challenge this model by demonstrating that the post-BE affect improves, which complicates the understanding of how affect regulation functions in BE. This complexity echoes discussions in the literature about whether BE primarily serves to alleviate negative emotions or if it can be reinforced through other mechanisms. Furthermore, other results, for example, Smith et al. [123], showed that when participants reported a greater instability in PA, they also reported higher overeating and LOC.

Mason et al. [126] found that both NAs and PAs, along with cognitive factors such as self-discrepancy, influence BE. The role of body dissatisfaction as a predictor of BE is well-supported by other studies [109], suggesting that both cognitive and affective variables must be considered in understanding BE. Moreover, the finding that adaptive emotion regulation strategies can prevent BE episodes [70] aligns with therapeutic approaches that emphasize improving emotional regulation skills in interventions for BED (e.g., ICAT and CBT).

Furthermore, the exploration of emotional variables showed that individuals with eating disorders like anorexia, bulimia, and BED exhibit distinct emotional profiles [148]. Evidence indicates that emotion regulation difficulties are pervasive across different types of eating disorders but differ in intensity, variability, and inertia, underscoring emotional dysregulation as a central feature of eating pathology [149]. Recent pilot data [150] further refined this picture by showing that normative guilt—guilt arising from perceived moral rule violations—was associated with binge eating and purging episodes, whereas altruistic guilt—stemming from concerns about harming others—predicted heightened interpersonal distrust. These findings suggest that distinct guilt subtypes may drive different symptom patterns in eating disorders. Incorporating such distinctions enriches the understanding of guilt’s multifaceted role and highlights the potential of EMA to capture these affective dynamics in real time, supporting the case for personalized interventions based on guilt profiles. Collectively, these insights emphasize the need for treatments that target emotion regulation skills and adapt to the specific emotional profiles of individuals.

Consistent with the growing body of literature on the EMA approach, affects and emotional dynamics are the most frequently studied topics. EMAs provide unique opportunities to model affects at both between-individual and within-individual levels, capturing fluctuations that traditional cross-sectional or long-term longitudinal designs may overlook.

Several studies in the review investigate the dynamics of dietary lapses and temptations, indicating their strong associations with environmental cues, emotional states, and psychological variables such as self-efficacy and stress [54,65,72,129,132,139]. This is consistent with behavioral models that describe lapses as critical moments that determine longer-term outcomes in weight control (e.g., Marlatt & Gordon’s Relapse Prevention Model). For instance, Goldstein et al. [92,129] highlighted that exposure to food cues and missed meals or snacks are common predictors of dietary lapses, reinforcing the idea that lapses often occur in contexts of high temptation or a reduced self-regulatory capacity. Furthermore, Crochiere et al. [132] found that urges to deviate from an eating plan, cravings, and alcohol consumption predict dietary lapses, which is consistent with findings from the literature on self-regulation failure and “what-the-hell” effects in dietary contexts [151]. Furthermore, obesity determinants, correlates, and consequences associated with overeating are particularly triggered in interpersonal situations, and especially for BED, it is important to capture the frequencies of eating that did not necessarily coincide with traditional mealtimes such as breakfast, lunch, snack, and dinner. In fact, event-contingent designs may be suitable to detect relevant moments. Interventions focusing on improving mindful awareness and acceptance, such as ACT [81,105], show promise in reducing the impact of NA on dietary lapses, reflecting broader evidence that mindfulness-based interventions can help mitigate overeating and enhance weight loss outcomes [106].

The included studies on food cravings emphasize the significant differences between individuals with obesity and healthy controls, particularly in terms of craving intensity, frequency, and control. These results resonate with other studies showing that food cravings are more frequent and intense in individuals with obesity and are often associated with emotional eating and poorer weight management outcomes [152]. The finding that mindful eating interventions can reduce craving-related eating and self-reported overeating behaviors [106,107] aligns with the growing body of literature advocating for mindfulness-based approaches to manage cravings. These interventions appear to weaken the link between negative moods and cravings, which has been identified as a significant challenge for individuals attempting to lose weight [153].

The prevalence of weight stigma and its impact on psychological well-being and eating behaviors has been well-documented. Studies reviewed here, such as Vartanian et al. [114] and Carels et al. [95,115], confirm that experiences of weight stigma, both internal and external, are pervasive and linked to Negative Affects and maladaptive coping behaviors. These findings are consistent with the broader literature that suggests that weight stigma exacerbates psychological distress and may perpetuate unhealthy eating behaviors [154].

The distinction between IWB and external stigma is particularly noteworthy, as it highlights the role of self-directed stigma in predicting maladaptive outcomes, such as emotional eating and exercise avoidance. This adds to the literature on internalized stigma, which suggests that the internalization of negative societal attitudes can be more damaging than the experience of overt discrimination [155].

As an emerging topic, the relationship between personality, personality pathology, and obesity is a relevant topic in contemporary psychopathology that could be further explored with EMA strategies.

Another important issue to consider is the integration of EMA into psychological interventions. This approach is gaining traction and can be effectively combined with routine outcome monitoring procedures, which have demonstrated significant effects in both controlled and naturalistic settings [156]. Routine outcome monitoring is becoming an increasingly popular strategy, offering new opportunities to bridge the gap between research and practice. In this context, only a few studies used EMA to support a psychological intervention [63,66,67,91,92,93].

Another promising area is the incorporation of behavioral and physiological processes through the use of sensors and biosensors. Multimethod measurements that combine both passive and active assessments can be highly valuable, leveraging the strengths of each approach. Alongside advancements in machine learning algorithms, the expansion of EMAs becomes more feasible, which is crucial for enhancing the personalization of potential treatments. Despite this, only four studies included physiological outcomes using sensors and biosensors.

Since most studies were conducted in the United States and other high-income Western countries, their findings may not generalize well to other contexts. Cultural and contextual factors significantly influence psychopathological conditions, including obesity. The rise in open-source platforms can help disseminate research methods to those with limited resources, such as researchers in low- and middle-income countries, reducing the bias toward WEIRD populations. Additionally, paying participants to enhance compliance, a common practice in these studies, should be considered when aiming to improve the external validity of future EMA research, as previously argued [157].

Finally, there are important aspects to discuss regarding the methodological design of the studies included in the present systematic review. Most of the studies were well-designed, employing advanced statistical methods, and published in high-impact journals. However, several studies did not report important information regarding the design or results. Lacking information undermined the quality of reporting as well as the transparency and as a consequence, the reproducibility and replicability of the study. Potential limitations concerning the quality of the studies seem to be related to the lack of clear guidelines and standards, which have only recently started to emerge [158]. Furthermore, in terms of data analysis, the majority of studies employed multilevel or hierarchical linear models [159]. When ANOVAs or Ordinary Least Squares (OLS) models were used instead of hierarchical models, their findings should be interpreted more cautiously, as these approaches do not account for data dependency. In intensive longitudinal studies like Ambulatory Assessments, where data is inherently nested, relying on such methods can lead to inaccurate representations of the data. Future studies should incorporate new modalities of data analysis, such as multilevel network analyses, and also include qualitative investigations to overcome the limitations of relying solely on self-report approaches. Moreover, most included studies did not provide an a priori power analysis to justify sample sizes. This issue is similar to those found in other areas of psychology. For instance, a recent review in the field of psychopathology revealed that only 2% of the studies included had reported a power calculation [158]. Performing sample size calculations for EMA studies is challenging, as it involves estimating various parameters that are often difficult to determine in advance without access to pilot data or prior studies that provide comprehensive model outputs. Unfortunately, such detailed reporting is frequently lacking, with random effects often excluded from articles and Supplementary Materials. Although there are published tutorials on how to conduct power analyses for EMA studies [160,161], their adoption appears to be quite limited.

### Strengths and Limitations

This is the first systematic review of the application of the EMA method in the context of overweight and obesity since its inception. We provided an overview of psychological and contextual predictors examined across EMA studies, highlighting differences in focus and identifying gaps for future research. Although there is currently no consensus on how to determine the quality of EMA studies reliably, we therefore adopted a quality appraisal tool, drawing on available checklists. Another strength is the choice to follow the principles of Open Science, including study pre-registration. The results of this review should be interpreted with certain limitations. First, the inclusion criteria excluded gray literature, such as dissertations and preprint repositories. As a result, there are some missed relevant studies. However, this exclusion was intended to ensure that only peer-reviewed articles were included, maintaining a high standard of rigor. Additionally, we limited our review to published, peer-reviewed articles in English, excluding those in other languages. Furthermore, the calculation of inter-rater agreement metrics (e.g., Cohen’s kappa) could improve the quality of the article. Finally, this review represents the first synthesis summarizing the literature on EMA studies for adults with obesity, and it is only a preliminary step; no definitive conclusions should be drawn. Future quantitative analyses, such as meta-analyses, should be conducted on the specific topics identified in this study. Some of the included studies are likely to have used overlapping samples. Finally, since most of the included studies involved White or Caucasian participants, future research should place greater emphasis on including ethnic minority groups to enhance the generalizability of the findings. For the same reason, a systematic review focused on adolescents could help validate and strengthen our results.

## 5. Conclusions

Overall, the findings of the present study suggest that EMA could be considered a powerful approach to capture psychological processes in populations with overweight and obesity. The reviewed studies align well with the existing literature in emphasizing the complex interplay of psychological, emotional, and environmental factors in overweight- and obesity-related behaviors. Future studies could benefit from integrating these various aspects to provide a more holistic understanding of obesity and inform more effective, multifaceted interventions. In particular, EMA could be incorporated into clinical practice by using smartphone-based prompts to monitor dietary intake, physical activity, mood, and contextual triggers in real time. By continuously monitoring eating episodes, physical activity patterns, emotional states, and environmental triggers, EMA data can inform the delivery of just-in-time adaptive interventions (JITAIs) that provide tailored prompts or coping strategies at the exact moment when maladaptive behaviors are most likely to occur. Such dynamic feedback systems could be incorporated into mobile health (mHealth) platforms and integrated into routine follow-up visits. This would enhance patient self-awareness, help identify high-risk situations, and foster a greater personalization of treatment goals. Moreover, aggregated EMA profiles may facilitate multidisciplinary collaboration, as dietitians, psychologists, and physicians can jointly interpret momentary data to align nutritional, behavioral, and pharmacological strategies. Future research should examine the feasibility, acceptability, and clinical effectiveness of EMA-based approaches across nutritional, behavioral, and psychological domains, while also considering scalability and integration within routine multidisciplinary care. The quality of future EMA studies could be improved by conducting a priori power analyses and better accounting for EMA missingness. Finally, by integrating EMA with personalized medicine, the healthcare system can promote proactive and targeted care that meets the specific needs of individuals in diverse contexts and settings, like obesity.

## Figures and Tables

**Table 1:** Quality assessment.

Author, Year	Quality 1—Rationale for the EMA Design	Quality 2—Whether an a Priori Power Analysis Had Been Conducted	Quality 3—Adherence to the EMAs	Quality 4—Treatment of Missingness	Total
Alabduljader et al., 2018 [76]	2	1	1	1	5
Ambwani et al., 2015 [99]	1	1	1	1	4
Bartholomay et al., 2024 [84]	3	1	2	3	9
Boh et al., 2016 [62]	2	2	2	1	7
Booker et al., 2024 [80]	1	1	1	1	4
Carels et al., 2001 [51]	2	1	1	1	5
Carels et al., 2004 [113]	3	1	2	1	7
Carels et al., 2019 [95]	1	1	2	1	5
Carels et al., 2019 [115]	1	1	1	1	4
Chwyl et al., 2023 [103]	2	1	2	1	6
Clark et al., 2022 [104]	1	3	1	2	7
Cnudde et al., 2024 [79]	2	3	3	2	10
Coffman et al., 2021 [105]	1	1	3	1	6
Crochiere et al., 2022 [132]	2	1	3	1	7
Dougherty et al., 2024 [102]	1	3	1	1	6
Dougherty et al., 2025 [56]	1	3	1	1	6
Emerson et al., 2018 [131]	1	1	2	1	5
Engel et al., 2009 [86]	2	1	1	1	5
Forester et al., 2023 [117]	2	1	1	1	5
Forester et al., 2024 [108]	3	1	3	1	8
Godfrey et al., 2019 [81]	2	1	1	1	5
Goldschmidt et al., 2012 [87]	2	1	2	1	6
Goldschmidt et al., 2014 [97]	2	1	2	1	6
Goldschmidt et al., 2014 [125]	1	1	2	1	5
Goldschmidt et al., 2017 [130]	1	1	2	1	5
Goldschmidt et al., 2018 [144]	1	1	2	1	5
Goldstein et al., 2018 [92]	2	1	2	2	7
Goldstein et al., 2018 [129]	2	2	3	3	10
Goldstein et al., 2022 [112]	2	3	2	3	10
Hagerman et al., 2023 [139]	2	1	2	1	6
Hagerman et al., 2024 [111]	1	1	3	2	7
Hilbert et al., 2007 [69]	2	1	2	1	6
Imes et al., 2021 [137]	1	1	1	1	4
Kalan et al., 2024 [120]	1	1	3	1	6
Keating et al., 2019 [74]	3	1	2	3	9
Keith et al., 2016 [89]	1	1	1	1	4
Kerver et al., 2025 [52]	3	1	3	1	8
Kornacka et al., 2021 [78]	1	1	1	1	4
Kuipers et al., 2025 [55]	2	1	2	1	6
Latner et al., 2013 [65]	1	1	1	1	4
Li et al., 2024 [121]	3	1	3	1	8
MacDonald et al., 2024 [75]	1	1	3	1	6
MacIntyre et al., 2021 [118]	1	1	2	1	5
Manasse et al., 2022 [138]	1	1	3	1	6
Margaryan et al., 2025 [59]	1	1	3	1	6
Mason et al., 2018 [106]	1	3	2	2	8
Mason et al., 2021 [135]	2	1	2	2	7
Mason et al., 2022 [128]	2	1	2	1	6
Mason et al., 2022 [126]	3	1	3	1	8
Mason et al., 2022 [126]	3	1	2	1	7
Mason et al., 2024 [122]	3	1	3	1	8
Mckee et al., 2014 [72]	3	1	1	1	6
Morales et al., 2025 [58]	3	1	2	1	7
Munsch et al., 2009 [67]	3	1	3	2	9
Munsch et al., 2012 [68]	3	1	3	1	8
Neal et al., 2025 [57]	2	1	3	1	7
Nechita et al., 2023 [77]	3	1	1	3	8
Olson et al., 2023 [98]	1	1	3	1	6
Parker et al., 2021 [101]	1	1	1	1	4
Peterson et al., 2020 [85]	2	3	3	3	11
Pollert et al., 2013 [88]	1	1	3	1	6
Potter et al., 2021 [147]	2	3	1	1	7
Ralph-Nearman et al., 2024 [134]	1	1	1	2	5
Rancourt et al., 2015 [143]	1	1	1	1	4
Roefs et al., 2019 [63]	3	1	3	1	8
Roordink et al., 2023 [64]	2	2	3	1	8
Roordink et al., 2025 [54]	2	2	3	1	8
Ruf et al., 2025 [60]	3	1	3	2	9
Sagui-Henson et al., 2021 [107]	2	3	3	3	11
Sala et al., 2021 [124]	1	3	3	2	9
Schaefer et al., 2020 [127]	3	1	2	3	9
Schaefer et al., 2021 [100]	3	1	3	1	8
Schaefer et al., 2023 [119]	3	1	2	2	8
Scherer et al., 2022 [90]	3	3	1	1	8
Schumacher et al., 2018 [140]	1	1	3	2	7
Seiferth et al., 2024 [71]	2	1	1	1	5
Smith et al., 2024 [123]	3	1	3	1	8
Srivastava et al., 2021 [93]	1	1	3	1	6
Srivastava et al., 2024 [109]	2	1	3	1	7
Stein et al., 2007 [133]	1	1	3	1	6
Svaldi et al., 2019 [70]	2	1	3	2	8
Thøgersen-Ntoumani et al., 2021 [73]	3	1	2	2	8
Unick et al., 2021 [82]	1	3	3	2	9
Vartanian et al., 2014 [114]	1	1	1	1	4
Wetzel et al., 2025 [53]	3	3	2	2	10
Wilkinson et al., 2024 [116]	3	3	3	1	10
Williams-Kerver et al., 2020 [94]	3	2	3	2	10
Williams et al., 2016 [83]	3	1	3	3	10
Wonderlich et al., 2024 [110]	1	1	3	1	6
	Weak: 35 (39.32%); Moderate: 28 (31.46%); Strong: 26 (29,21%)	Weak: 70 (78.65%); Moderate: 5 (5.61%); Strong: 14 (15.73%)	Weak: 24 (26.96%); Moderate: 27 (30.33%); Strong: 38 (42.69%)	Weak: 63 (70.78%); Moderate: 17 (19.10%); Strong: 9 (10.11%)	
Legend: 1: Weak, 2: Moderate; and 3: Strong					

## Data Availability

No new data were created or analyzed in this study.

## Supplementary Materials

The following supporting information can be downloaded at: https://www.mdpi.com/article/10.3390/jpm15110526/s1, File S1: Study Characteristics; Table S1: PRISMA 2020 Checklist.
